# Differential diagnosis of synchronous double primary squamous cell carcinomas of the esophagus and occult primary oropharynx through HPV testing: A case report

**DOI:** 10.1097/MD.0000000000042243

**Published:** 2025-05-02

**Authors:** Hee Kyung Kim, Yong-Pyo Lee, Hongsik Kim, Yaewon Yang, Jihyun Kwon, Ki Hyeong Lee, Seung-Myoung Son, Hye Sook Han

**Affiliations:** a Department of Internal Medicine, Chungbuk National University Hospital, Cheongju, Republic of Korea; b Department of Internal Medicine, Chungbuk National University College of Medicine, Cheongju, Republic of Korea; c Department of Pathology, Chungbuk National University Hospital, Cheongju, Republic of Korea; d Department of Pathology, Chungbuk National University College of Medicine, Cheongju, Republic of Korea.

**Keywords:** esophageal cancer, HPV, NGS, oropharyngeal cancer

## Abstract

**Rationale:**

Esophageal and head and neck cancers often coexist due to field cancerization, which causes multiple squamous cell carcinomas (SCCs) in the upper aerodigestive tract. However, guidelines for the differential diagnosis and optimal treatment of synchronous esophageal and head and neck SCC are lacking. This study highlights the diagnostic and therapeutic challenges of these coexisting malignancies.

**Patient concerns:**

A 52-year-old man presenting with dysphagia was diagnosed with esophageal SCC, and an incidental retropharyngeal tumorous lesion was found during staging.

**Diagnoses:**

Biopsy revealed SCC in both lesions. To differentiate between metastatic disease and a second primary SCC, immunohistochemistry (IHC) and next-generation sequencing (NGS) were performed. The retropharyngeal SCC showed strong p16 staining, indicating human papilloma virus (HPV)-related origin, while the esophageal SCC was p16-negative. NGS revealed distinct genetic profiles for each lesion, confirming synchronous double primary SCCs.

**Interventions:**

The patient underwent definitive concurrent chemoradiotherapy for both SCCs.

**Outcomes:**

The patient achieved a complete response for both lesions and remains recurrence-free 1 year after treatment.

**Lessons:**

This case underscores the importance of HPV testing and/or NGS in patients with multiple SCCs in the upper aerodigestive tract to accurately differentiate primary cancers from metastases.

## 1. Introduction

Multiple primary malignancies are frequently detected in patients with esophageal cancer, and squamous cell carcinoma (SCC) of the head and neck is the second most common type of malignancy when primary esophageal SCC is present.^[1,2]^ Multiple SCCs in the upper aerodigestive tract are explained by the concept of field cancerization, which accounts for the association of multiple tumors in this region.^[3,4]^ Synchronous double primary SCCs of the esophagus and head and neck are often incidentally identified during staging work-ups with computed tomography (CT) and/or positron emission tomography (PET)-CT scans.^[5]^ The management of the synchronous SCCs of the esophagus and head and neck is not well established, and the prognosis of each SCC is generally poor.^[6,7]^ Additionally, given that SCCs of the esophagus and head and neck usually have morphologically similar histology, it is difficult to distinguish double primary SCCs or synchronous metastases from each SCC. Furthermore, when head and neck cancer is an occult primary SCC with only metastatic neck lymph nodes detected, it is often misdiagnosed as metastasis from esophageal SCC.

Herein, we report the favorable course of a patient with the synchronous double primary SCCs of the esophagus and occult primary oropharynx, using immunohistochemistry (IHC) staining and next-generation sequencing (NGS) to differentiate between the synchronous SCCs.

## 2. Case presentation

**Figure 1.**
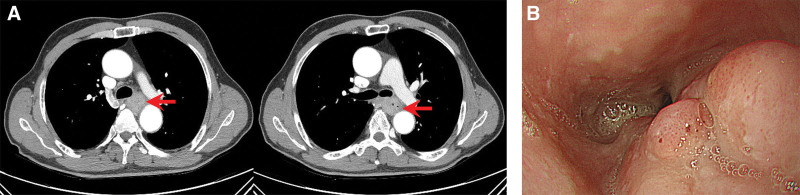
Computed tomography and esophagogastroduodenoscopy. (A) Computed tomography of the chest showed enhanced wall thickening and luminal narrowing of the mid-esophagus. (B) Esophagogastroduodenoscopy revealed an eccentric ulceroinfiltrative mass in the lower thoracic esophagus at 26 cm from the incisors.

**Figure 2.**
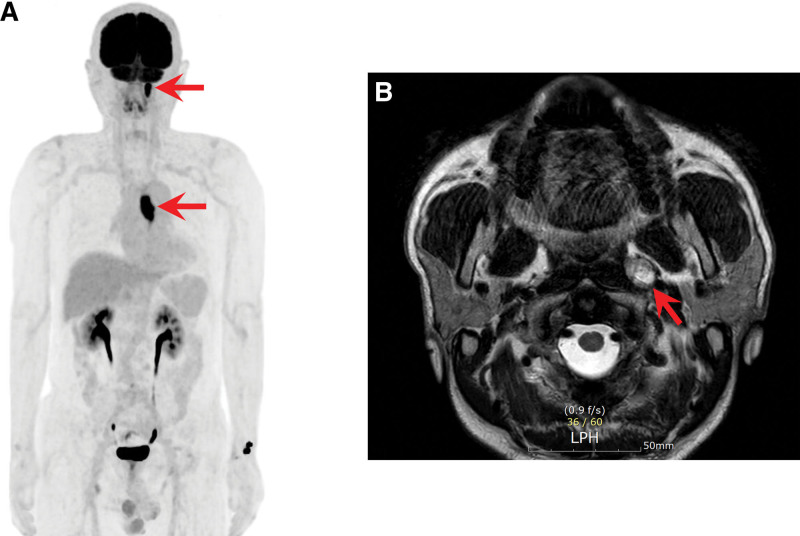
Positron emission tomography and magnetic resonance imaging. (A) Positron emission tomography showed ^18^F-FDG uptake in the mid-thoracic esophagus and left retropharyngeal area. (B) Magnetic resonance imaging of the neck showed a focal, well-defined mass in the left retropharyngeal area. FDG = fluorodeoxyglucose.

A 54-year-old man presented to the gastroenterology department with progressive dysphagia of 1-month duration. He was previously healthy and had no known co-morbidities. He reported needing to drink more water to aid swallowing over the past month and being unable to swallow even water the day before his visit. He has smoked 1 pack of cigarettes daily for the past 20 years and denied alcohol consumption. On admission, he was hemodynamically stable, and his initial laboratory results including complete blood cell counts, liver function tests, and coagulation studies were within the normal ranges. A CT scan of the chest and abdomen revealed focal irregular enhancing wall thickening and luminal narrowing in the mid-esophagus, along with subtle wall thickening in the lower trachea and left main bronchus, suggesting esophageal cancer with possible invasion to the trachea and/or main bronchus (Fig. 1A). Esophagogastroduodenoscopy showed an ulceroinfiltrative mass in the middle thoracic esophagus, located 26 cm from the incisors (Fig. 1B). The biopsy of the esophageal mass confirmed SCC. Bronchoscopy revealed no endobronchial lesion. A PET-CT scan was performed for staging work-up and revealed hypermetabolic lesions in the mid-thoracic esophagus (maximum standardized uptake value [SUVmax = 20.4]) and a focal hypermetabolic lesion in the left retropharyngeal area (SUVmax = 14.4; Fig. 2A). The retropharyngeal lesion was asymptomatic and diagnosed incidentally. Magnetic resonance imaging of the neck revealed a focal, well-defined mass in the left retropharyngeal space (Fig. 2B). No tumorous lesions suspicious for primary head and neck cancer were observed during the comprehensive head and neck examination, which included fiberoptic evaluation of the nasopharynx, oropharynx, hypopharynx, and larynx.

**Figure 3.**
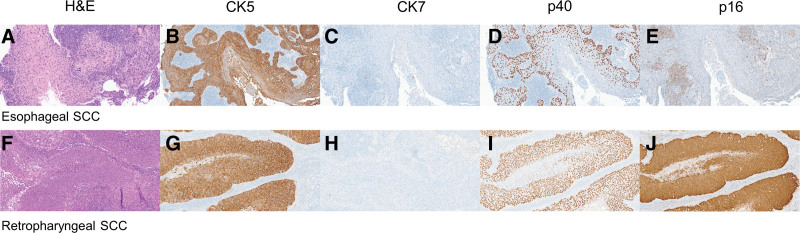
Pathologic findings of the tumors in the esophagus and retropharyngeal lymph node. Histologic examination of the esophagus and left retropharyngeal lymph node revealed squamous cell carcinoma (A, F: hematoxylin and eosin, ×100). Immunohistochemistry showed tumor cells in the esophagus and left retropharyngeal lymph node positive for cytokeratin 5 (×400) and p40 (×400), but negative for cytokeratin 7 (×400) (B–D, G–I). The esophageal SCC was negative for p16 (E), but the staining was strongly positive in the left retropharyngeal SCC (J). SCC = squamous cell carcinoma.

We conducted a multidisciplinary discussion with surgeons from the ear, nose, and throat and cardiothoracic surgery departments, as well as a medical oncologist, radiation oncologist, radiologist, pathologist, and the patient. As a result, we planned and performed a left retropharyngeal LN dissection to confirm double primary head and neck cancer. The histology results of the retropharyngeal LN also showed SCC, which could not be distinguished from metastasis of esophageal SCC on hematoxylin and eosin staining (Fig. 3A, F). Therefore, IHC staining of the SCCs of the esophageal mass and retropharyngeal LN was performed. Staining for cytokeratin 5 (CK5), CK7 and p40 as markers of SCC demonstrated similar expression in the esophageal and retropharyngeal SCCs (Fig. 3B–D, G–I). The esophageal SCC was negative for p16 (Fig. 3E). However, the retropharyngeal SCC was highly positive for p16 (Fig. 3J). NGS was performed on each SCC using the Oncomine^TM^ Comprehensive Assay Plus, Level II. The esophageal SCC showed an *RB1* deletion and point mutations in *TP53*, *APC*, and *CREBBP*, while the retropharyngeal SCC displayed amplifications in the *PIK3CA*, *PIK3CB*, and *CCND1* genes.

The final diagnosis of the patient was cT3N0Mx, esophageal SCC and pTxN1M0, human papilloma virus (HPV)-mediated (p16+) oropharyngeal SCC according to the American Joint Committee on Cancer. He was diagnosed with localized synchronous double primary SCCs of the esophagus and occult primary oropharynx. After a thorough multidisciplinary discussion of treatment options, the patient elected to undergo definitive concurrent chemoradiotherapy. The regimen consisted of cisplatin (60 mg/m² on days 1 and 22) and 5-fluorouracil (1000 mg/m² on days 1–4 and days 22–25), accompanied by radiotherapy. The planned radiotherapy doses were 5000 cGy to the left retropharynx and oropharynx and 5040 cGy to the esophagus. This targeted approach aimed to address both primary malignancies while minimizing toxicity. During treatment, the patient tolerated chemotherapy and radiotherapy without significant interruptions or severe adverse events. One month after completing the radiotherapy, a follow-up CT scan showed tumor shrinkage, and 6 months later, follow-up CT scan and esophagogastroduodenoscopy showed a complete response. Over 1 year of the follow-up, including routine CT scans and endoscopic assessments, no recurrence was observed in either the esophageal or oropharyngeal regions.

## 3. Discussion and conclusions

Here, we present a patient with esophageal SCC and HPV-mediated (p16+) occult oropharyngeal SCC that was diagnosed incidentally and treated successfully. Concurrent chemoradiotherapy was administered for esophageal and oropharyngeal SCC, and the treatment outcome was favorable. In a previous study, 35.1% of patients with esophageal cancer were found to have double primary head and neck cancer.^[5]^ The patient in the current study also had synchronous double primary head and neck cancer, and the primary tumor had resolved, presenting only with metastatic neck LN as occult oropharyngeal SCC. This second tumor was an incidental finding and initially difficult to distinguish from neck LN metastasis of esophageal SCC versus a double primary head and neck SCC. To the best of our knowledge, this report provides the first guidance for differentiating metastases of esophageal SCC from double primary head and neck SCC using p16 IHC staining and NGS testing.

In recent years, HPV has emerged as a significant risk factor of head and neck cancer.^[8,9]^ HPV-associated oropharyngeal cancer demonstrates a more favorable prognosis compared to other types of head and neck cancer. Consequently, determining the HPV status of the tumor is an essential component in the diagnosis of head and neck cancer and can serve as an important method to demonstrate distinct cancers when accompanied by esophageal cancer.^[10,11]^ Furthermore, tumors with histologically similar morphology are at risk of being misdiagnosed as metastasis of 1 tumor. In such cases, NGS testing can help identify them as distinct double primary cancer.

As demonstrated in this case, when esophageal SCC and head and neck SCC are diagnosed concurrently, appropriately treating both SCCs can be challenging. In a recent meta-analysis, definitive chemoradiotherapy and esophagectomy were equally effective as initial treatments for potentially resectable esophageal SCC.^[12]^ However, surgery combined with radiation or chemoradiation showed a survival benefit compared to definitive chemoradiation therapy alone.^[13]^ Since surgical resection of head and neck cancer and esophageal cancer can significantly decrease quality of life, definitive concurrent chemoradiotherapy, which allows organ preservation, may be a suitable treatment option for patients with both types of cancer. Identifying double primary cancers in this patient enabled delivery of the ideal treatment for each cancer through concurrent chemoradiotherapy. In addition, a multidisciplinary team, comprising medical, surgical, and radiation oncologists, as well as pathologists and radiologists, was incorporated into the care plan for diagnosing and treating this patient. A multidisciplinary team approach has been shown to improve survival rate in patients with multiple primary cancers including esophageal cancer,^[14]^ and in the current case, a multidisciplinary team approach enabled optimal diagnosis and treatment.

A potential limitation of this study is that NGS may not be readily accessible in all clinical settings, particularly in resource-limited environments. Furthermore, while the patient has remained recurrence-free for 1 year, longer follow-up is necessary to confirm the durability of the treatment outcomes and to evaluate potential late-onset treatment-related side effects.

In conclusion, this case underscores the importance of precision diagnostics, including HPV testing and NGS, in distinguishing synchronous primary SCCs from metastases and optimizing treatment strategies. The successful outcomes highlight the feasibility of definitive concurrent chemoradiotherapy in achieving disease control for patients with similar presentations.

## Author contributions

**Conceptualization:** Hee Kyung Kim, Hye Sook Han.

**Data curation:** Yong-Pyo Lee, Hongsik Kim.

**Investigation:** Hee Kyung Kim, Seung-Myoung Son.

**Methodology:** Yaewon Yang, Jihyun Kwon.

**Resources:** Seung-Myoung Son, Hye Sook Han.

**Supervision:** Seung-Myoung Son, Hye Sook Han.

**Validation:** Ki Hyeong Lee, Hye Sook Han.

**Writing – original draft:** Hee Kyung Kim.

**Writing – review & editing:** Hye Sook Han.
